# Fine motor skills disorders in the course of Wilson's disease

**DOI:** 10.4103/0972-2327.48849

**Published:** 2009

**Authors:** Peter Albrecht Günther, Hans-Juergen Kühn, Thomas Villmann, Wieland Hermann

**Affiliations:** Clinic of Neurology, University of Leipzig; 1 Clinic of Neurology, Institute of Laboratory Medicine, Clinical Chemistry and Molecular Diagnostics, University of Leipzig; 2 Clinic for Psychosomatic Medicine, University of Leipzig; 3 Department of Neurology, Paracelsus Clinic, Zwickau

**Keywords:** Fine motor skills, Wilson's disease, writing sample

## Abstract

**Objectives:**

Fine motor skills disorders belong to the neurological manifestation of Wilson's disease. The aim of this study is to investigate if fine motor performance changes during the course of the disease and with therapy.

**Methods:**

In 15 neurological patients with Wilson's disease, severity of neurological symptoms was assessed with a neurology score. A test battery consisting of the hand writing of a test sentence, lines of “double-I” and retracing a circle was carried out for analysis. By means of a computer-aided analysis of the patient's handwriting, 10 kinematic parameters of the writing trace were calculated. These parameters were determined once at the very beginning of the study and then again after 7 years.

**Results:**

Improvement of clinical symptoms was observed after onset of therapy only within the first 2 years. In contrast to the standard population, a reduced degree of automation could be detected both at the beginning and at the end of the 7-year interval. There was no significant change in 8 out of the 10 kinematic parameters during the observation period, 2 deteriorated.

**Discussion:**

The absence of a significant increase in fine motor disturbances proves, on the one hand, the efficacy of the therapy regime applied. On the other hand, the end point of a possible reversibility had been reached. A computer-aided analysis of the patient's handwriting allows for a sensitive detection of the “functional scar” in the extrapyramidal control and can subsequently prompt a timely correction of therapy in case of progression.

## Introduction

Wilson's disease is an autosomal recessive disorder of the hepatic copper metabolism leading to a biliary copper excretion dysfunction.[1,2] The corresponding gene defect is located on the long arm of chromosome 13 in the 13q14-21-Locus.[3,4] Up to now, approximately 250 different mutations of the *ATP7B*-gene have been identified.[5] Most mutations are rare, only a limited number is common and specific to a certain group of the population. Mutation H1069Q is the most common one in Europe with an allele frequency of 65%.[6–11]

According to the presence of either hepatic or neurological and, as the case may be, also psychiatric symptoms, a division into either hepatic or neurological types of the disease is established.

Basal ganglionic and cerebellar disorders are characteristics of the neurological course of the disease. On the onset of symptoms, different manifestations, such as rigor, tremor, dystonia, ataxia, choreoathetosis, gait disturbances, hypersalivation, dysarthria and fine motor skills disturbances present in great variability.[12–16]

Fine motor performances require an intact basal ganglionic and cerebellar control. Automated handwriting skills, in particular, as movements relevant to our day-to-day lives, depend on exact motor control. In the neurological type of Wilson's disease the cerebellar part is primarily affected, whereas the basal ganglionic part of this control is affected to a much lesser degree.[17–19]

The morphological correlation for this is the presence of basal ganglionic lesions (putamen, substantia nigra, nucleus ruber) and a cerebellar atrophy apparent in MRI diagnostics.[20–23] In T2-weighted MRI the so-called “status spongiosus” is characteristic of the toxic impact of copper.

Early therapy can result in almost complete reversibility of extrapyramidal symptoms. Former studies have shown that after 2 years of therapy, persisting symptoms are irreversible.[17,24,25] Therefore, early initiation of therapy is crucial to limiting the severity of persisting disturbances.[26]

The following study examines the fine motor deficit in the generation of the writing trace during the course of therapy of Wilson's disease. The aim of the study is to find proof if the fine motor deficit evident in the patient's writing trace can either be stabilised or even decreased under sufficient copper-removing therapy over the course of 7 years.

## Materials and Methods

### Patients

For the recruitment of data, 15 neurological patients (10 women, 5 men, median at first examination 44 years, youngest 31 years, oldest 63 years) were examined. An intravenous radio copper test[27] confirmed that all patients were in fact suffering from Wilson's disease. They had all been in long-term drug therapy for at least 2 years prior to the first examination in 1999.

After diagnosis, all patients were put on a progressive chelation therapy with either D-penicillamine (DPA) or trientine, initially administered in small doses. During the subsequent period, up to 1999, 6 patients were changed to a therapy with zinc salt and 9 patients received adjusted doses of chelating agents on the basis of follow-up examinations of both the clinical symptoms and the copper metabolism which were carried out annually. After 1999, none of the patients were subjected to a further change of therapy, only some drug dosage adjustments were made.

The following neurology score [Table 1] depicts the characteristics of neurological symptoms, first at the time of diagnosis as initial baseline degree of severity, then again at the time of the first fine motor examination in 1999 and finally after an interval of 7 years in 2006.[28]

**Table 1:** Neurology score[11]

Extrapyramidal motor symptoms	Evaluation of disturbance in a Neurology score	
Fine motor disturbance		
Dysdiadochokinesis	Very severe	5
Rest and postural tremor	Severe	4
Abnormality of muscular tone	Medium	3
Ataxia	Light	2
Brady-/Hypokinesis		
Gait disturbance		
Only dysarthria		1
Nothing		0

### CS-Writing test

**Figure 1:**
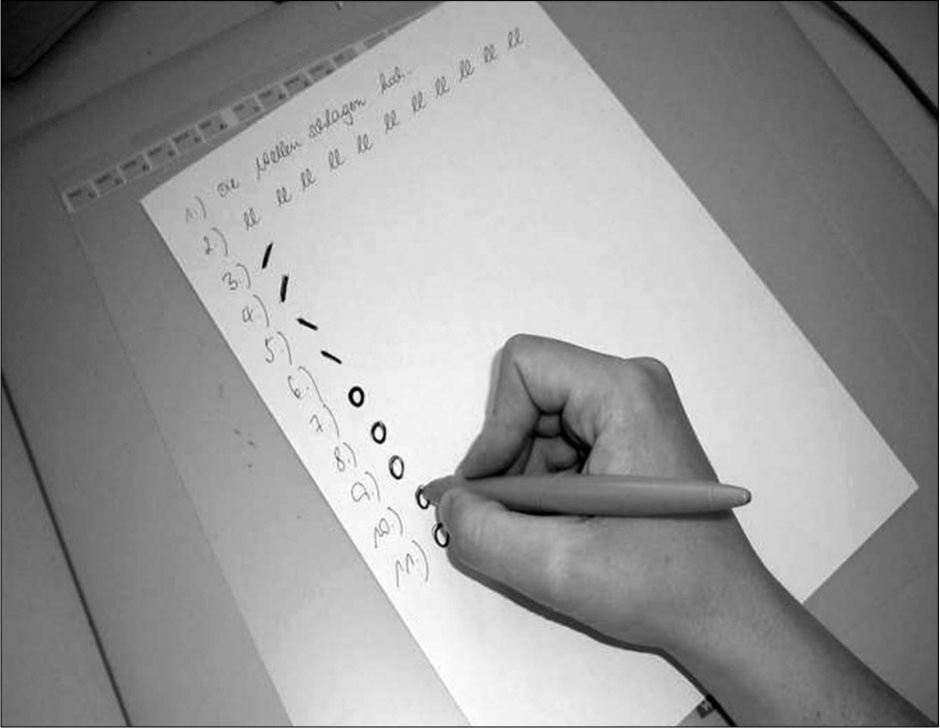
Graphic tablet

A graphic tablet (company of Borgemann, Dortmund) allowed for a documentation of the writing sample under natural conditions [Figure 1]. With this tablet, the location of the tip of the ballpoint pen was registered at a chronological resolution of 200 data points per second. The graphical and statistical evaluation of the registered writing trace as well as the calculation of the kinematic parameters were carried out applying the “CS” computer program (MedCom Publishing Company, Munich).[29]

In the present study, writing a test sentence (“Die Wellen schlagen hoch.” – “The waves are rising high”.), writing lines of “double-l” for 10 s as well as drawing and repeatedly retracing a circle for 3 s were the set tasks up for analysis. The patients were asked to carry out the tasks swiftly in the sense of an automated writing movement.

For the purpose of facilitating the statistical evaluation, the writing trace was divided into up- and down-strokes, which formed the basis of further calculation (segment analysis).[29–31]

**Figure 2:**
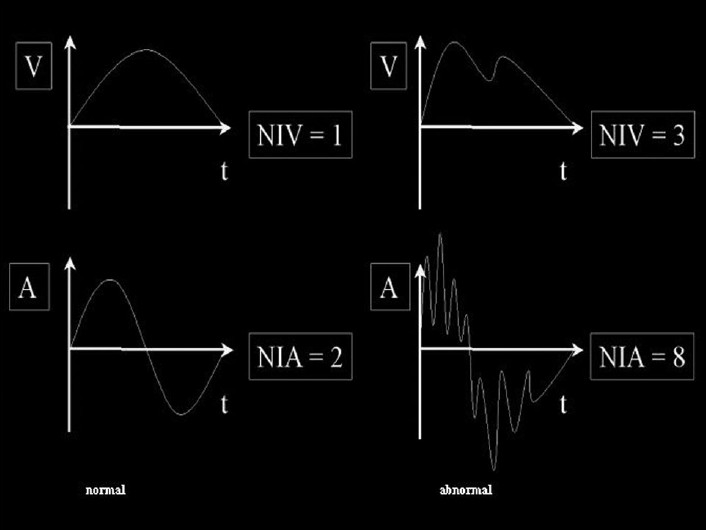
Velocity (V) and acceleration (A) profile of a normal movement segment next to an abnormal one (schematical)

The number of inversions in velocity within one up- or down-stroke respectively (NIV) and the frequency (number of up- and down-strokes per second) were the kinematic parameters of the writing trace determined here. The corresponding acceleration profile (number of inversions in acceleration, NIA) is at a maximum before the maximum velocity and at a minimum after the maximum velocity [Figure 2].[32] The standard values of 156 test volunteers are contained in Table 2. Maximum or minimum values for NIV, NIA, frequency and required time for sentence were consulted for reference.[30]

**Table 2:** Standard values of the kinematic parameters of the writing trace

	NIV*	NIA*	Frequency*	Time required*
Test sentence	1.3 ± 0.21	2.4 ± 0.17	5.2 ± 0.31 Hz	9.6 ± 1.31 sec
	maximum < 2.0	maximum < 3.0	minimum > 2.0	maximum < 15 sec
“II II II”	1.2 ± 0.19	2.2 ± 0.19	5.6 ± 0.42 Hz
	maximum < 2.0	maximum < 3.0	minimum > 2.0
Circle	1.3 ± 0.17	2.5 ± 0.23	5.4 ± 0.36 Hz
	maximum < 2.0	maximum < 3.0	minimum > 2.0

* : values outside maximum or minimum are regarded as confirmed abnormal[30]

### Statistics

By subjecting the resulting data matrix of fine motor skills parameters to the Kolmogorow-Smirnov test, a normal distribution could be ruled out. Consequently, the median was hence used as localization criterion. For the purpose of comparing the two cohorts, dependent random samples had to undergo the Wilcoxon test, whereas the Mann-Whitney U test was applied to all independent random samples. The level of significance was set at *P* < 0.05.

## Results

### Neurology score

The study substantiated improvements of neurological symptoms in all patients compared with their respective pathology at the time when therapy was first initiated. The files of the follow-up examinations carried out at intervals of 1 or 2 years documented that symptoms continued to improve for the first two years of therapy. Thereafter, symptoms remained constant until the time of the first examination (1999) that was carried out in the context of the present study. The median degree of improvement is 2 points in the neurology score. In the following 7-year observation interval, no further clinical-neurological improvements or changes occurred [Table 3].

**Table 3:** Patient chart with therapy regime applied, neurology score and fine motor parameters of the writing trace

Patient	Time	Therapy	Neurology score	Test sentence				Double-L			Circle		
						
				TS NIV	TS NIA	TS frequency	TS time	LL NIV	LL NIA	LL frequency	C NIV	C NIA	C frequency
1 WA	Initial	DPA	4										
	1999	DPA	3	6.69	9.00	1.37	50.00	8.69	9.15	1.42	6.00	7.00	1.57
	2006	DPA	2	5.45	9.54	1.11	18.86	10.69	14.67	0.75	9.5	10.33	0.86
2 WK	Initial	DPA	5										
	1999	DPA	3	12.17	9.73	1.18	50.00	8.08	8.54	1.39	5.50	7.67	1.64
	2006	DPA	3	4.16	7.05	1.56	19.54	5.76	8.85	1.29	5.67	9.20	1.29
3 HS	Initial	DPA	5										
	1999	DPA	3	3.29	3.13	2.83	10.79	2.69	4.15	2.78	1.73	2.00	4.06
	2006	DPA	3	2.28	3.20	2.61	15.34	2.42	3.22	2.86	10.00	13.00	0.97
4 BM	Initial	DPA	3										
	1999	DPA	1	1.54	2.72	3.66	9.96	3.84	6.79	2.04	2.00	7.40	2.07
	2006	DPA	1	1.67	2.68	2.96	12.66	2.33	4.11	2.14	2.33	6.00	1.73
5 JS	Initial	DPA	3										
	1999	DPA	2	5.71	8.21	1.74	50.00	5.67	7.13	1.76	2.00	3.00	3.02
	2006	DPA	2	7.10	10.26	1.02	19.59	5.71	8.20	1.23	2.11	4.75	1.82
6 Bl	Initial	DPA/trientine	4										
	1999	Trientine	1	2.20	3.53	3.02	11.56	4.56	6.50	1.74	2.43	3.29	2.96
	2006	Trientine	1	1.48	2.74	3.05	14.25	2.22	4.94	1.70	3.25	6.71	1.64
7 KH	Initial	DPA	4										
	1999	Trientine	0	2.54	2.71	3.05	11.30	2.91	3.77	2.38	1.18	1.36	4.14
	2006	Trientine	2	1.84	2.71	2.71	16.81	2.26	3.67	2.25	2.33	3.73	2.19
8 BR	Initial	DPA	4										
	1999	Trientine	2	2.24	3.17	3.17	9.46	3.72	5.16	2.59	1.60	1.80	4.15
	2006	Trientine	2	1.90	2.83	3.11	11.41	2.12	3.95	2.30	3.50	6.20	1.31
9 LE	Initial	DPA	2										
	1999	Trientine	0	1.49	2.29	4.18	10.30	1.06	2.56	3.32	1.36	1.60	3.91
	2006	Trientine	1	1.91	2.52	2.73	17.42	1.29	1.70	4.02	3.57	7.67	1.31
10 KM	Initial	DPA	3										
	1999	Zinc	0	1.74	2.41	3.78	9.13	1.31	2.44	3.31	1.40	3.00	3.86
	2006	Zinc	0	1.81	2.66	3.18	11.81	1.91	3.70	2.28	1.75	6.43	1.67
11 LR	Initial	DPA	3										
	1999	Zinc	1	2.89	4.17	2.47	14.39	2.78	6.67	1.93	1.00	3.00	3.73
	2006	Zinc	1	2.15	3.35	2.29	19.03	2.74	4.93	1.73	1.33	3.73	2.48
12 LD	Initial	DPA	5										
	1999	Zinc	3	9.76	11.50	1.11	50.00	20.00	17.40	0.72	8.33	17.00	1.25
	2006	Zinc	3	8.42	10.09	0.98	18.43	11.14	12.54	0.84	13.33	18.00	0.67
13 RA	Initial	DPA	4										
	1999	Zinc	0	1.67	2.73	2.99	11.20	2.28	3.00	2.58	3.80	6.00	1.98
	2006	Zinc	0	1.55	2.32	3.03	13.19	1.72	2.91	2.44	2.33	5.75	1.73
14 SA	Initial	DPA	5										
	1999	Zinc	4	2.47	3.65	3.29	9.59	3.00	4.11	2.82	2.33	3.50	3.20
	2006	Zinc	3	2.33	3.06	2.96	10.65	2.25	3.30	2.48	2.23	4.33	2.40
15 VC	Initial	DPA	5										
	1999	Zinc	2	2.44	4.73	2.80	13.74	3.57	5.19	2.29	5.50	6.00	1.62
	2006	Zinc	2	2.02	2.76	2.97	19.35	1.69	2.94	2.68	6.40	9.50	1.13
median	Initial		4.00										
	1999		2.00	2.47*	3.53*	2.99	11.30	3.57*	5.19*	2.29	2.00*	3.29*	3.02
	2006		2.00	2.02*	2.83	2.73	16.81*	2.26*	3.95*	2.25	3.25*	6.43**	1.64**
Significant differences of median value (p-value)				0.16	0.24	0.11	0.065	0.07	0.17	0.247	0.053	0.006	0.00
				n	n	n	n	n	n	n	n	s	s

n: not significant, TS: test sentence, C: circle,

* : significant deterioration betweeen 1999 and 2006,

s: significant, LL: double-L,

* : pathological median compared to normal value,

### CS writing test

At the time of the first examination in 1999, the kinematic parameters of all three tests (test sentence, “double-l” and circle) show a disturbance in the fine motor control of the writing motor function in contrast to the standard population. Raised median values of both the NIV and NIA are indicative of this, whereas the median frequency of the writing trace (up-and down-strokes) still remains within the lower normal range at 2.99 Hz (test sentence), 2.29 Hz (“double-l”) and 3.02 Hz (circle). The time required to complete the test sentence does not exceed the set time limit. A total of 6 out of the 10 median values of the kinematic parameters determined are abnormal [Table 3].

Eight out of the 10 kinematic parameters present no significant change at the end of the 7-year interval. Over the course, the median NIA (6.43) and median frequency (1.64 Hz) during the “circle test” show substantial deviations (deteriorations) compared to 1999 (*p* < 0.05) and are abnormal. The NIV median (3.25) is also raised in this test but does not differ significantly from the value determined in 1999.

During the test sentence, both the median NIV parameter (2.02) and the median time required to write the test sentence (16.81 sec) deviate from the norm. The “double-l test” continues to present abnormal values of the NIV (2.26) and NIA (3.95), while the frequency still remains within the norm. In contrast to the control group, 7 out of 10 fine motor parameters show abnormal median values [Table 3].

### Comparison of medication

The 15 patients were divided into two therapy cohorts according to the type of subsequent long-term therapy that followed their initial therapy regime with chelating agents: 6 patients on zinc salts and 9 patients under further therapy with DPA or trientine [Table 3].

Between 1999 and 2006, the therapeutic effect that had been achieved with initial therapy of DPA or trientine was maintained under both the chelating agent therapy and the changeover to treatment with zinc salts. Neither cohort showed any changes in the neurology score.

The difference between the 10 kinematic parameter values of 1999 and 2006 was calculated for the purpose of reviewing possible effects of therapy on the patient's fine motor performance. The resulting values were attributed to the two therapy groups (chelating agents vs. zinc) and tested for significant differences by carrying out the Mann-Whitney U test. In 9 out of the 10 parameters examined, these differential values were in fact independent of the therapy regime applied. Only the difference of the writing frequency during the “circle test” was significant (*p* < 0.05), and is much higher under DPA therapy.

## Discussion

The automated control of movements as regards time and tonus is dependent on an intact reciprocally inhibiting interconnection of the basal ganglia and the cerebellar coordination.[33–36] These control systems correspond to the exposed vulnerable structure in the context of a copper intoxication of the central nervous system.[37] Both the morphological irregularities in MRI[38,39] and the traceable metabolic deviations in the [^18^F] FDG-PET[40–43] substantiate structural and functional lesions in the basal ganglia and the cerebellum in the presence of Wilson's disease. Abnormal findings after β-CIT-SPECT [^123^I]β-CIT and [^123^I]IBZM IBZM-SPECT supply evidence of deficits in the dopaminergic nigrostriatal system in neurological Wilson's disease patients.[44,45]

Extrapyramidal motor symptoms clinically improve well under copper-removing therapy. The significant reversible part of extrapyramidal symptoms is clinically quantified in the neurology score applied here. Irrespective of the severity of initial symptoms, the improvement noted was 2 points in the applied score. This aspect reflects the reversible share of the functional impairment. In correlation to this, a partial regression of MRI lesions and a functional recovery of the nigrostriatal system could be observed.[40,46]

Disturbances of automated fine motor movements, however, cannot be subjected to exact clinical assessment. They continue to be characteristic of the neurological course of Wilson's disease as an expression of an irreversible basal ganglionic cerebellar disorder, whereas other symptoms do, in fact, subside. Our handwriting skills are of great relevance to our daily lives. The generation of the writing trace and the presence of subtle disturbances cannot be assessed visually so that a graphic tablet is required to record these subtleties. The computer-aided analysis captures the fine motor performance with mathematical exactness.

During the 3 tests carried out in the present study, a total of 10 kinematic parameters were determined in the context of fine motor skills follow-up examinations. In all patients, all of the parameters are affected by abnormal deviations of varying degree compared to normal values. At the 1999 examination, a median of 6 parameters pathological increased to a median of 7 in 2006. This data substantiates a disorder of the fine motor control and correlates with the low degree of automation in the patient's handwriting skills after long-term drug therapy of more than 2 years.

The follow-up examination 7 years later shows a persistence of the fine motor skills disturbances. Compared to the results of the initial examination in 1999, 8 out of the 10 parameters did not change significantly. Only 2 parameters were substantially different and had deteriorated by the end of the 7-year examination interval. The general final conclusion is that the fine motor damage profile of the present cohort of neurological patients does not present any relevant dynamic development under the therapy regime applied.

On the one hand, the fine motor skills of patients, who had already been under drug therapy for at least 2 years, did not improve during the examination period. Following an initial convalescence of symptoms, the ultimate point of possible reversibility under therapy had been reached 7 years previously. The persistence of symptoms can therefore be interpreted as a functional scar of the basal ganglionic and cerebellar control. Therefore, we can conclude that an attenuation of fine motor disorders is only possible within the first few months of starting therapy. This is consistent with the fact that any reversibility of symptoms in the morphological and imaging diagnostics remains merely partial.

A more efficient early therapy, i.e., with tetrathiomolybdate, could have a favourable influence on the reversibility of symptoms.[47] In any case, this assumption remains speculative and should therefore be examined under this very aspect.

However, the clinical stability of symptoms (neurology score) over a period of 7 years marks both long-term drug therapies – with zinc salts as well as with the chelating agents trientine and D-penicillamine – as equally efficient. This fact is further substantiated by the fine motor analysis of the patient's handwriting skills at subsequent follow-up examinations. Both therapy cohorts achieve similar results in 9 out of the 10 parameters during the 7-year period.

The decisive factor influencing the extent of the persisting fine motor disorders is primarily an early initiation of therapy rather than the actual choice of copper-removing medication administered, provided that a negative copper balance is thus constantly attainable.

The study confirms that a sensitive registration of the fine motor disorders of the patient's handwriting skills is made possible by applying the CS system. The continuation of therapy serves to prevent progression of the disease and can be monitored by checking the fine motor control of the patient's handwriting.
